# Contralateral Ear Occlusion for Improving the Reliability of Otoacoustic Emission Screening Tests

**DOI:** 10.1155/2014/248187

**Published:** 2014-01-12

**Authors:** Emily Papsin, Adrienne L. Harrison, Mattia Carraro, Robert V. Harrison

**Affiliations:** ^1^Auditory Science Laboratory, Neuroscience and Mental Health Program, The Hospital for Sick Children, 555 University Avenue, Toronto, ON, Canada M5G 1X8; ^2^Institute of Biomaterials and Biomedical Engineering, University of Toronto, Toronto, ON, Canada M5S 1A1; ^3^Department of Otolaryngology-Head and Neck Surgery, University of Toronto, 190 Elizabeth Street, Toronto, ON, Canada M5G 2N2

## Abstract

Newborn hearing screening is an established healthcare standard in many countries and testing is feasible using otoacoustic emission (OAE) recording. It is well documented that OAEs can be suppressed by acoustic stimulation of the ear contralateral to the test ear. In clinical otoacoustic emission testing carried out in a sound attenuating booth, ambient noise levels are low such that the efferent system is not activated. However in newborn hearing screening, OAEs are often recorded in hospital or clinic environments, where ambient noise levels can be 60–70 dB SPL. Thus, results in the test ear can be influenced by ambient noise stimulating the opposite ear. Surprisingly, in hearing screening protocols there are no recommendations for avoiding contralateral suppression, that is, protecting the opposite ear from noise by blocking the ear canal. In the present study we have compared transient evoked and distortion product OAEs measured with and without contralateral ear plugging, in environmental settings with ambient noise levels <25 dB SPL, 45 dB SPL, and 55 dB SPL. We found out that without contralateral ear occlusion, ambient noise levels above 55 dB SPL can significantly attenuate OAE signals. We strongly suggest contralateral ear occlusion in OAE based hearing screening in noisy environments.

## 1. Introduction

Audiometric testing in general is best carried out in a low noise environment. Indeed most clinical testing is done in sound attenuating booths, where background noise levels are typically below 20 dB SPL (for frequencies of audiometric interest). For performing behavioral (pure tone and speech audiometry) and physiological tests (auditory evoked potentials and OAEs) the focus has been on maintaining a good signal to noise ratio for the test signals presented. The issue addressed in the present study pertains not to the test ear but to the contralateral ear that may or may not be occluded. In neonatal or newborn hearing screening with OAEs most protocols do not specify any occlusion or plugging of the nontest ear (e.g., [1–11]). However, such screening tests are routinely carried out in a noisy hospital or clinic environments. Newborn babies may be screened in patient's rooms, clinical areas, or a neonatal intensive care unit (NICU), where ambient sound levels can be as high as 60–70 dB SPL (e.g., [12–16]). The American Academy of Pediatrics recommends that sound levels in an NICU should not exceed 45 dB, but most often this is not the case. Indeed a review by Konkani and Oakley reveals that ambient noise levels in typical NICUs can exceed 80 dB SPL [16].

It is now well established that OAEs—discovered by Kemp in 1978 [17]—are suppressed or modulated by acoustic signals presented to the contralateral ear. The role of the olivocochlear neural efferent system in inhibiting outer hair cell activity is well understood [18–24]. The consequent modulation of the outer hair cell mechanics and their contribution to OAE generation are the basis of clinical tests of the contralateral OAE suppression reflex [25–36].

The question posed in the present study is do ambient noise levels, typical of OAE screening environments, suppress OAEs in the test ear by stimulation of the contralateral, nonoccluded ear? In a sense the answer is already known in that numerous studies (as referenced above) have utilized contralateral sound stimuli to enable OAE suppression, which have stimulus levels that are similar to those of ambient noise. Furthermore, work including that by our own group [35] has clearly shown that OAE suppression is not a reflex with a defined threshold response. The efferent system enables OAE suppression with contralateral stimuli over a wide range of stimulus intensities. In other words acoustic signal levels constantly influence the system. We have chosen to “model” the situation of hearing screening testing in environments with different level of ambient noise.

## 2. Materials and Methods

### 2.1. Subjects and OAE Measurements

We tested 6 young adult females (18–24 yrs.) with normal audiograms and robust OAEs (signals above noise, in the normal range and repeatable). OAE recordings were made in each individual ear (*N* = 12). Two OAE measurement methods were used. Transient evoked (TE) OAEs (ILO88 Otodynamics, Hatfield, UK) and distortion product (DP) OAEs (Vivo 600DPR; Vivosonic, Toronto, Canada). In each of the 4 acoustic environments (described below) TEOAE and DPOAE measures were repeated 3 times with and without occlusion of the contralateral ear. The ear canal was occluded with a standard memory foam earplug, and a circumaural headphone shell was also worn to achieve a combined attenuation greater than 40 dB. We measured TEOAEs to click stimuli (ILO88 default mode) and quantified using the average dB response. DPOAEs were measured in the form of a DPgram; 2*f*1-*f*2 signal levels as a function of *f*2 frequency (0.25–6 kHz; e.g., Figure 3). These DPgrams were quantified by simple average of emission levels at all test frequencies.

### 2.2. Acoustic Environments

(i) Control experiments were carried out in a sound attenuated booth (single wall ACO) with ambient sound levels below 25 dB SPL (100 Hz–16 kHz). (ii) Experiments were also made in the open laboratory environment, where ambient noise level was approximately 45 dB SPL. (iii) A study was made in noise-augmented environment in which white noise generation was adjusted to give an overall ambient noise level of 55 dB SPL. (iv) A recorded babble/shopping mall sound sample was used to provide a 55 dB SPL ambient noise that was more dynamic in character than the white noise augmented environment. In other words this background noise had significant temporal and spectral fluctuations. All acoustic signal levels were measured in free field at the level of the subject's head using a calibrated (B&K 4230, 94 dB 1 kHz) sound meter (Larson Davis 831) with half-inch condenser microphone (PCB Piezotronics). We used a linear (nonweighted) mode with a 100 Hz–16 kHz bandwidth.

### 2.3. Data Analysis

For each acoustic condition, TEOAE and DPOAE signals with and without plugging of contralateral ear are compared with a two tailed, paired Student's *t*-test, after confirmation of normal data distribution with Kolmogorov and Smirnov analysis.

## 3. Results

### 3.1. TEOAE Results

**Figure 1:**
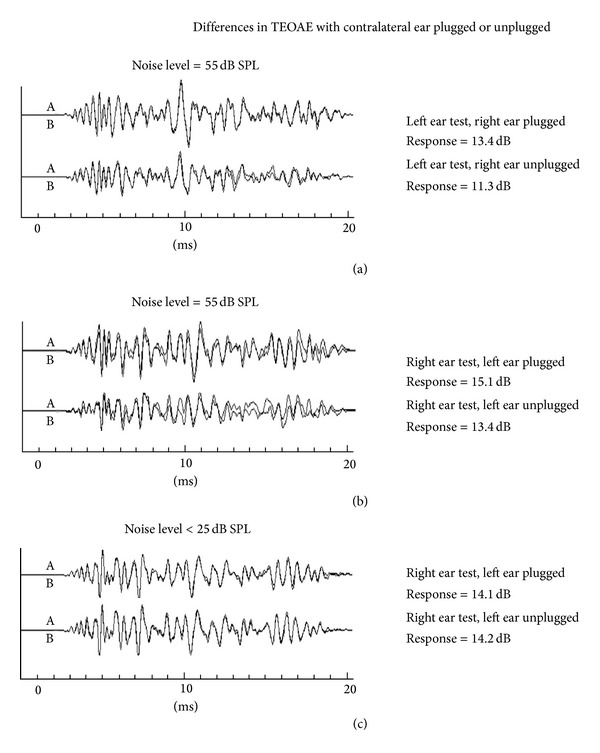
Differences in TEOAE wave forms (ILO88 format) measured with contralateral ear canal plugged or open, in 55 dB SPL ambient noise level ((a) and (b)) versus noise levels <25 dB SPL (c). Data shown are from one subject.

Figure 1 illustrates OAE waveforms evoked by broadband click stimuli in the ILO88 (Otodynamics) format; results are from one subject. Each data pair is a record made with and without contralateral ear plugging. The upper two data records were made in the environment with a 55 dB SPL ambient noise level. Note the attenuation of the wave forms in the nonplugged ear canal condition. In both cases the TEOAE response is decreased by almost 2 dB. The lower traces show control records in the sound booth; contralateral ear occlusion does not alter TEOAE response.

**Figure 2:**
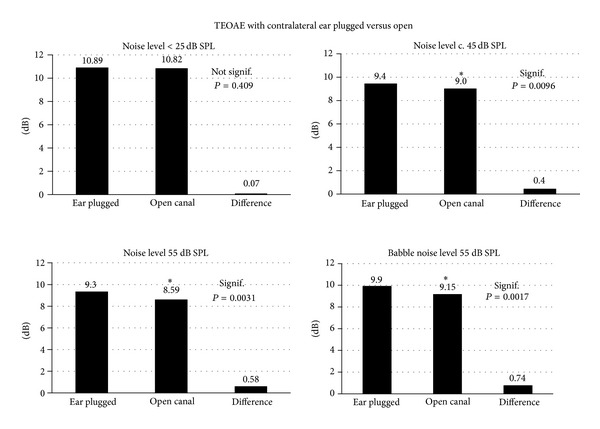
Plots of average DPOAE levels and the difference recoded with contralateral ear occluded or open (*N* = 6 subjects). Significance of the difference (paired Student's *t*-test) is indicated.

Table 1 lists, for all 6 subjects, the TEOAE levels (average of 3 repeat recordings) for the contralateral ear plugged and nonplugged conditions. The upper panel shows records made in the sound booth with ambient noise levels <25 dB SPL. There are no significant differences between contralateral ear plugged versus open ear canal conditions. Significance and *P* values of paired *t*-test results are listed. The lower panels show comparisons in sound environments with noise levels at 45 dB SPL and in (white) noise and babble noise augmented environments (55 dB SPL). In the 45 dB SPL environment three subjects have statistically significant differences in OAE level with versus without opposite ear plugging. In the 55 dB SPL ambient noise environments all but one subject show significant differences between TEOAE levels with and without opposite ear occlusion.  Figure 2 shows pooled subject data for each sound environment. Overall there is a significant difference in TEOAE levels for environments with ambient noise levels of 45 dB and above.

### 3.2. DPOAE Results

**Figure 3:**
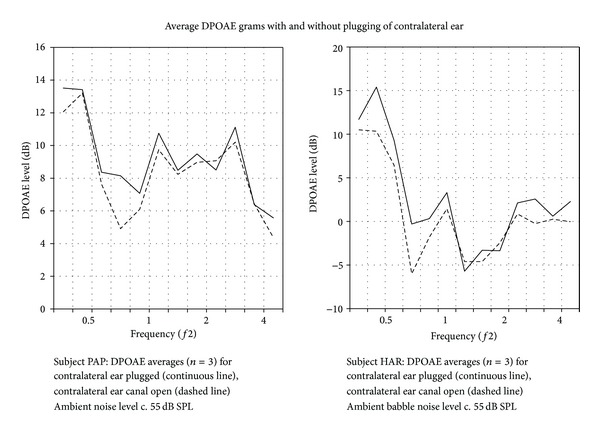
Example DPOAE (2*f*1-*f*2) versus frequency (*f*2) plots, DPgrams, for two subjects measured with (solid lines) and without (dashed curves) contralateral ear canal occlusion. Measurements were made in an environment with an ambient noise level of 55 dB SPL. DP grams shown are an average of three sequential recordings.

**Figure 4:**
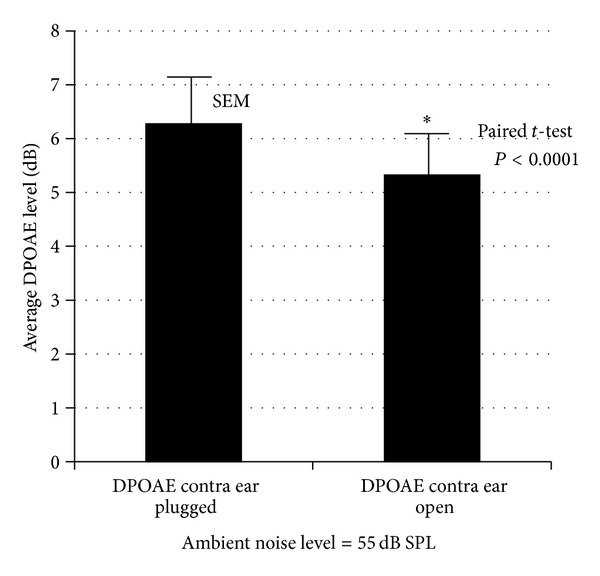
Average DPOAE level changes between contralateral ear open versus occluded conditions, measured in an environment with an ambient noise level of 55 dB SPL. Significant difference as indicated by paired Student's *t*-test.

Figure 3 shows DPgrams for two subjects measured in an environment with ambient noise at 55 dB SPL. In each case the solid lines indicate DPOAE level measured with contralateral ears plugged versus unplugged (dashed lines). Note the suppression caused by the environmental noise, especially between 0.5 and 1 kHz, where the decrease in DPOAE level amounted up to 3 dB.  Table 2 shows data from all 6 subjects. The 55 dB ambient environmental noise results in a significant contralateral suppression in only some subjects. However, it will be noted that the subjects with a significant suppression effect are those with an initially higher level DPOAE (subject list in Table 2 is ordered according to DPOAE level). Furthermore, the *P* values for the paired *t*-test are mainly low hinting of an effect. Indeed an analysis of pooled results graphed in Figure 4 shows a very significant effect (*P* < 0.0001) of the 55 dB SPL environmental noise.

## 4. Discussion

There has been some considerable attention paid to the issue of ambient noise in environments in which OAE screening tests are carried out. The main concerns however have related to the test ear rather than the contralateral ear. Thus there is concern about the signal-to-noise ratio in the test ear that has to be high for getting a valid OAE response [37, 38]. The authors are unaware of studies that have considered the effects of ambient noise on the contralateral ear. As previously mentioned, there are no provisions or recommendations to use occlusion of the contra lateral ears in screening testing, and thus the contralateral suppression effects on test ear OAEs is an issue. The effects of contralateral acoustic stimulation on OAEs have been extensively documented in experimental studies, animal models, and clinical research. It is surprising therefore that these effects have not been seriously considered in newborn hearing screening protocols that employ OAE measures.

In the present study, we have tested the hypothesis that moderate levels of environmental noise can suppress OAE responses by activation of the olivocochlear efferent system. In the present study a level of 55 dB SPL has a significant effect. Given that most hospital ward and clinic environments have ambient noise levels higher than 55 dB SPL we conclude that, unless the untested ear is occluded, there will almost certainly be a suppression effect. It should be noted that clinical diagnostic OAE testing is almost always carried out in a low noise environment, typically in a sound attenuating booth. Here the problem of contralateral ear stimulation is negligible. However, in neonatal hearing OAE screening the availability of a sound booth or even a quiet environment is not a reality. It has been suggested that the olivocochlear efferent system is not fully matured or operational in a neonatal human subject, and therefore the precaution of occluding the contralateral ear is unnecessary. It has been reported that in some species efferent innervation is one of the final stages of cochlear maturation [39–41]. In the mouse, an altricious species, efferents do not fully connect with outer hair cells until postnatal day 20 [42]. However, the human is a precocious species with a much more mature peripheral auditory system at birth. There is some evidence that continued maturation of contralateral OAE suppression continues for some weeks after term birth [43]. However, a number of authors report that OAE suppression reflexes can be recorded in at term [36, 44, 45].

The results of this present study indicate that with a 55 dB ambient noise OAE levels can be attenuated by as much as 3 dB. It could be argued that such small attenuations will be of little significance in a screening test. However, it should be noted that this level of ambient noise is very low compared with that in a typical NICU or hospital clinic environment. Furthermore, 3 dB is a significant level change when the original OAE signal level may be of a similar order of magnitude. Will small OAE attenuations make a difference in a pass/refer (fail) screening paradigm? We suggest that it will definitely lead to more false positive results, and that means increasing parent anxiety and further healthcare costs.

## 5. Conclusion

In OAE screening tests, a nonoccluded contralateral ear will be stimulated by ambient environmental noise. Noise levels above 55 dB SPL can significantly suppress OAEs in the test ear and lead to false positive results. Such inaccuracy can be avoided by occlusion of the contralateral ear canal.

## Figures and Tables

**Table 1:** TEOAE data from six subjects comparing OAE levels with contralateral ear occluded versus open. *P* values of paired Student's *t*-test results and significance are indicated.

Subject	TEOAE (dB) contra ear plugged	TEOAE (dB) contra ear open	Difference (dB)	*P* value	Significance
Noise level <25 dB SPL					
PAP	14.21	14.11	0.1	0.42	NO
ALL	9.42	9.3	0.12	0.629	NO
GLU	13.1	13.4	−0.3	0.471	NO
LAR	10.35	10.05	0.3	0.46	NO
HAR	5.08	4.86	0.21	0.15	NO
SKL	13.2	13.18	0.016	0.6109	NO

Noise level c. 45 dB SPL					
PAP	14.59	13.62	0.96	0.003	**YES**
ALL	9.6	8.35	1.25	0.044	**YES**
GLU	13.22	12.28	0.93	0.112	NO
LAR	9.12	8.72	0.4	0.093	NO
HAR	5.75	5.6	0.13	0.604	NO
SKL	12.12	11.65	0.46	0.0004	**YES**

Noise level 55 dB SPL					
PAP	13.98	12.88	1.1	<0.0001	**YES**
ALL	8.55	7.7	1.05	0.0085	**YES**
GLU	13.55	11.9	1.65	<0.0001	**YES**
LAR	8.78	7.9	0.88	0.151	NO
HAR	5.75	5.0	0.21	0.021	**YES**
SKL	12.28	11.92	0.366	0.0197	**YES**

Babble level 55 dB SPL					
PAP	13.63	12.62	1.02	0.0113	**YES**
ALL	9.47	8.41	1.057	0.0036	**YES**
GLU	13.55	12.15	1.4	0.0002	**YES**
LAR	9.43	8.33	1.1	0.009	**YES**
HAR	5.95	5.38	0.56	0.035	**YES**
SKL	13.35	12.95	0.4	0.0015	**YES**

**Table 2:** DPOAE data from six subjects indicating OAE level recorded with and without occlusion of the contralateral ear in four different ambient sound environments. Results of Student's *t*-test are indicated.

Subject	DPOAE (dB) contra ear plugged	DPOAE (dB) contra ear open	Difference	*P* value	Significance
Noise level <25 dB SPL					
PAP	10.76	10.77	−0.014	0.937	NO
DAV	9.73	9.66	0.07	0.88	NO
LAR	6.9	6.59	0.3	0.378	NO
ALL	4.38	4.3	0.087	0.625	NO
HAR	3.94	3.62	0.32	0.234	NO
GLU	2.8	1.64	1.165	0.105	NO

Noise level c. 45 dB SPL					
DAV	12.56	12.39	0.168	0.457	NO
PAP	8.78	8.56	0.23	0.467	NO
LAR	6.9	6.37	0.53	0.351	NO
HAR	5.48	4.97	0.51	0.66	NO
ALL	5.21	4.58	0.63	0.323	NO
GLU	2.32	2.18	0.148	0.805	NO

Noise level 55 dB SPL					
DAV	10.96	10.22	0.74	0.0861	**almost **
PAP	8.73	7.95	0.777	0.0995	**almost**
LAR	7.5	6.02	1.48	0.001	**YES**
HAR	5.68	4.37	1.31	0.153	NO
ALL	4.14	3.21	0.92	0.117	NO
GLU	3.23	2.39	0.84	0.2537	NO

Babble level 55 dB SPL					
PAP	10.12	9.13	0.99	0.0061	**YES**
LAR	7.27	5.7	1.56	0.0481	**YES**
DAV	6.96	5.5	1.46	0.0054	**YES**
HAR	4.49	4.03	0.45	0.417	NO
ALL	4.62	3.39	1.23	0.165	NO
GLU	1.62	1.96	−0.34	0.627	NO
